# Upregulated TRAIL and Reduced DcR2 Mediate Apoptosis of Decidual PMN-MDSC in Unexplained Recurrent Pregnancy Loss

**DOI:** 10.3389/fimmu.2020.01345

**Published:** 2020-06-30

**Authors:** Congcong Li, Xiaoxin Zhang, Xiaomin Kang, Chao Chen, Feng Guo, Qiaohong Wang, Aimin Zhao

**Affiliations:** ^1^Department of Obstetrics and Gynecology, Renji Hospital, School of Medicine, Shanghai Jiao Tong University, Shanghai, China; ^2^Shanghai Key Laboratory of Gynecologic Oncology, Shanghai, China; ^3^Department of Reproductive Medical Center, The First People's Hospital of Yunnan Province, Kunming, China

**Keywords:** polymorphonuclear myeloid-derived suppressor cell, TRAIL, TRAIL receptor, apoptosis, unexplained recurrent pregnancy loss

## Abstract

Myeloid-derived suppressor cells (MDSC), especially polymorphonuclear MDSC (PMN-MDSC), accumulate in maternal-fetal interface during pregnancy and are involved in the maintenance of immune tolerance. Decreased PMN-MDSC is associated with pregnancy complications such as unexplained recurrent pregnancy loss (URPL). In the present study we showed decreased PMN-MDSC in the URPL group compared with the normal pregnancy (NP) group, and PMN-MDSC was the major subset of MDSC in human decidua with potent immune suppression activity. We then performed gene expression profile and found that human decidual PMN-MDSC in the NP and URPL groups showed different gene and pathway signature, including apoptosis. Apoptosis of decidual PMN-MDSC was mediated by TNF-related apoptosis–induced ligand (TRAIL) in a Caspase 3 dependent manner. TRAIL was expressed in decidua and upregulated in decidua of the URPL group. Notably, of all the membrane TRAIL receptors, only DcR2 was down-regulated in PMN-MDSC in the URPL group. *In vitro* experiment demonstrated that DcR2 blockade sensitized PMN-MDSC to TRAIL-mediated apoptosis. Together, these data indicate that increased TRAIL and reduced DcR2 on PMN-MDSC sensitize PMN-MDSC response to TRAIL-induced apoptosis in the URPL group, which is responsible for decreased accumulation of PMN-MDSC in URPL.

## Introduction

Recurrent pregnancy loss (RPL), defined as two or more failed pregnancies, occurs in 5% pregnancies and about 50% of all RPL are idiopathic, which is defined as unexplained RPL (URPL) (1, 2). The pathogenesis of URPL is poorly understood and maternal-fetal immune dysfunction is considered to be one major cause. Maternal-fetal immune tolerance depends on intricate interactions of the immune system (3), and recent reports indicate a role of myeloid-derived suppressor cells (MDSC) in maintenance of maternal-fetal immune tolerance (4–6).

MDSC are recently identified as heterogeneous cell populations of myeloid origin with potent immunosuppressive activity (7, 8). In human, MDSC are defined as HLA-DR^−/low^CD11b^+^CD33^+^, and can be further categorized into two major subsets: CD33^bright^CD14^+^CD15^−^ monocytic MDSC (M-MDSC) and CD33^dim^CD14^−^CD15^+^ polymorphonuclear MDSC (PMN-MDSC) (8–10). Accumulation of MDSC occurs in a lot of physiological and pathological conditions, such as cancer, infection, autoimmune disorders, obesity, and pregnancy (7). Immune suppressive activity is the hallmark of MDSC. The most prominent immune regulatory factors of MDSC include Arginase 1, reactive oxygen species (ROS), prostaglandin E2, nitric oxide synthase, and immune checkpoints (11, 12). In different contexts MDSC suppress the immune response via different mechanisms (7). MDSC are involved in the maintenance of immune tolerance of pregnancy by inhibiting cytotoxic T cells activation, suppressing NK cells killing activities and regulating regulatory T cells (5, 6, 13, 14). Decreased MDSC has been associated with URPL and depletion of MDSC in murine pregnancy model can lead to implantation failure or embryo loss (6, 14, 15). Several studies have reported potential mechanisms of MDSC expansion during pregnancy in healthy women. Estrogen or progesterone can facilitate expansion and activation of MDSC (15, 16). Fetal-derived factor HLA-G also plays a role in PMN-MDSC accumulation via STAT3 pathway stimulation (17). Moreover, CXCR2/CXCL1 axis, which is also related to PMN-MDSC infiltration into tumor, promotes PMN-MDSC recruitment to the decidual microenvironment (18). Nevertheless, little is known about transcription features and cell fate of MDSC in normal pregnancy (NP) and URPL. Reagents targeting MDSC survival have been demonstrated to be effective for cancer treatment in both murine models and human participants (19–21). Understanding the cell fate of decidual MDSC is critical for developing better therapeutic approaches for pregnancy complications such as URPL.

In this study, we showed PMN-MDSC was the most abundant MDSC subset in decidua and only PMN-MDSC, not M-MDSC, decreased in decidua isolated from patients with URPL. Furthermore, we found apoptosis of decidual PMN-MDSC was activated in the URPL group. Increased TRAIL expression, together with reduced DcR2 in decidual PMN-MDSC, played an important role in excessive apoptosis of PMN-MDSC in URPL.

## Materials and Methods

### Study Participants

From June 2018 to December 2019, a total of 33 women with clinical NP and 23 women with URPL were enrolled in the study. The demographic characteristics of participants in the URPL group and the NP group are concluded in Supplemental Table 1. The fetal heartbeat of the NP group was verified by ultrasound before elective termination of pregnancy. Patients with URPL were enrolled in the URPL group if they fitted the following criteria: (1) two or more previous spontaneous abortions; (2) absence of uterine malformation; (3) normal karyotype of parents and abortus; and (4) absence of endocrine, metabolic, autoimmune diseases, thrombophilia, or infection; (5) Fetal heartbeat had ceased or never detected. Decidual tissues from 6 to 9 weeks were harvested immediately after surgery under sterile conditions and washed in cold PBS to remove blood and fetal tissues. Decidual tissues were frozen in liquid nitrogen for protein or RNA extraction. For immunohistochemistry, decidual tissues were fixed in formalin and embedded in paraffin for preservation. Peripheral blood was collected from healthy non-pregnant donors. The study was approved by the Human Research Ethics Committee of Renji Hospital and written informed consent was obtained from all participants.

### Cell Isolation

Single-cell suspensions were obtained by homogenizing tissues in PBS in the gentleMACS Dissociator (Miltenyi Biotec, Germany) with gentleMACS program B and C. Cell suspensions were strained through a 70-μm strainer and subsequently through a 40-μm strainer. Afterwards, the cells were washed with PBS and isolated using Ficoll density gradient (GE healthcare, USA). Mononuclear cells were harvested from the interphase. Cells were used immediately after isolation for phenotypic characterization and functional analysis. For sorting of decidual PMN-MDSC, decidual mononuclear cells were labeled with: CD11b-APC (BioLegend, USA), HLA-DR-FITC (BioLegend, USA) and CD15-PE (BD Bioscience, USA). PMN-MDSC were sorted as CD11b^+^HLA-DR^−^CD15^+^ using FACS Aria II (BD Bioscience, USA). In experiments involving survival analysis, CD15 MicroBeads (Miltenyi Biotec, Germany) and MACS sorting were used for separation of PMN-MDSC. To isolate CD3^+^ T cells for T cell suppression assay, peripheral blood mononuclear cells (PBMCs) of healthy donors were labeled with CD3 MicroBeads (Miltenyi Biotec, Germany) and sorted. Purity was >90% as confirmed by flow cytometry.

### Analytical Flow Cytometry

Fc receptor blocking solution (BioLegend, USA) was added prior to staining. The following antibodies were used in this study: CD45-APC-H7 (BD Bioscience, USA), CD45-Percp-Cy5.5 (BD Bioscience, USA), CD33-PE-Cy7 (BioLegend, USA), CD11b-FITC (BioLegend, USA), CD11b-BV421 (BD Bioscience, USA), CD11b-APC (BioLegend, USA), HLA-DR-BV421 (BD Bioscience, USA), HLA-DR-FITC (BioLegend, USA), CD14-PE (BD Bioscience, USA), CD15-APC (BD Bioscience, USA), CD15-BV510 (BioLegend, USA), CD3-FTIC (BioLegend, USA), IFN-γ-PE-Cy7 (BioLegend, USA), CD4-APC (BioLegend, USA), CD8-PE (BioLegend, USA), CD16-PE (BD Bioscience, USA), CD56-PE-Cy7 (BD Bioscience, USA), Fas-APC (BD Bioscience, USA), CD261-APC (BioLegend, USA), CD262-PE (BioLegend, USA), CD263-PE (eBioscience, USA), and CD264-PE (R&D Systems, USA). FVD eFluor 780 (eBioscience, USA) was used to identify dead cells for excluding them from the analysis. Flow cytometry data were acquired with LSRFortessa (BD Bioscience, USA) or Beckman Coulter FC500 (Beckman, USA), and were analyzed using FlowJo software (BD Bioscience, USA). Positive subpopulations were identified by comparing stained samples with Fluorescence minus one (FMO) controls.

### T Cell Suppression Assay

CD3^+^ cells were labeled with carboxyfluorescein diacetate succinimidyl ester (CFSE) (BD Bioscience, USA) according to the manufacturer's instructions and cultured with PMN-MDSC isolated from decidual tissues at a ratio of 2:1 or 6:1 in 96-well plates. CD3^+^ cells were stimulated with 10 μg/mL pre-coated anti-CD3 (OKT3, BioLegend, USA) and 1 μg/mL soluble anti-CD28 (CD28.2, BioLegend, USA). After 3.5 days of incubation, cells were resuspended in PBS for flow cytometry. For T cell secretion suppression assay, unlabeled CD3^+^ cells were used in the T: MDSC cell co-culture system. After 3.5 days, leukocyte activation cocktail with BD Golgiplug (BD Bioscience, USA) was added into the culture system for 5 h. Afterwards, the cells were harvested for the analysis of intracellular cytokine expression. Cells were cultured at 37°C in humidified air with 5% CO_2_ in RPMI 1640 (HyClone, USA) supplemented with 10% heat inactivated fetal bovine serum (FBS) (Gibco, USA) and 1% penicillin/streptomycin (Gibco, USA).

### Gene Expression Profile Analysis

The Human Whole Genome OneArray HOA 7.1 (Phalanx Biotech Group, China) was used to examine the whole-genome expression profiles of sorted PMN-MDSC from three women of the NP group and three women of the URPL group. Total RNA was extracted using Trizol Reagent (Invitrogen, USA) according to the manufacturer's instructions. The RNA integrity number (RIN) was 7–10. Cy5-labeled aRNA was hybridized and scanned on a G2505C Agilent Microarray Scanner (Agilent Technologies, USA) with Agilent 0.1 XDR software. Heatmap analysis was performed using R and a fold change of >1.5 was considered to be significant. Gene set enrichment analysis (GSEA) including GO and KEGG pathway was carried out using GSEA 4.0.1 and gene sets were obtained from the MSigDB database v7.0 (22, 23). The complete data were deposited in NCBI Gene Expression Omnibus with accession number GSE139180.

### Survival Assay

PMN-MDSC isolated from decidual tissues of normal pregnancy were cultured at 37°C in humidified air with 5% CO2 in RPMI 1640 (HyClone, USA) supplemented with 10% heat inactivated fetal bovine serum (FBS) (Gibco, USA), 1% penicillin/streptomycin (Gibco, USA) and 5 ng/mL recombinant GM-CSF (R&D Systems, USA). Decidual PMN-MDSC were treated with FasL (10, 100 ng/mL, BioLegend, USA), TRAIL (10, 100 ng/mL, Gibco, USA) or DR5 agonist Bioymifi (10, 50 μM, Selleck, USA) for 24 h and then were collected. In some experiments, PMN-MDSC were preincubated with anti-human DcR2 Ab (10 μg/mL, R&D Systems, USA) for 1 h before exposed to TRAIL. Apoptosis of PMN-MDSC was tested using activated Caspase-3-PE (BD Bioscience, USA) staining or Annexin V Apoptosis Detection Kit (BD Bioscience, USA) according to the manufacturer's instructions.

### Real-Time Quantitative RT-PCR

Total RNA was extracted from decidual tissues with TaKaRa MiniBEST Universal RNA Extraction Kit (Takara, Japan) according to the manufacturer's instructions. Concentration of RNA was measured by NanoDrop ND-1000 (Thermo Fisher Scientific, USA). mRNA was synthesized into cDNA using PrimeScript RT Reagent Kit (Takara, Japan). FasL, TRAIL and GAPDH were amplified through qRT-PCR using SYBR Premix Ex Taq II (Takara, Japan) with QuantStudio Dx Real-Time Instrument (Life Technologies, USA). For clinical samples, relative gene expression was calculated with 2^−Δ*CT*^ method normalized to GAPDH. The sequences of primers were listed in Supplemental Table 2.

### Western Blot

Decidual samples were homogenized, incubated with radio-immuno precipitation assay (RIPA) lysis buffer (Thermo Fisher Scientific, USA) with protease inhibitors (Sigma-Aldrich, USA) for 30 min on ice. Total protein extracts were obtained after centrifuging at 12,000 g for 15 min at 4°C. Protein concentrations were measured using a bicinchoninic acid assay (BCA) assay kit (Beyotime Biotechnology, China). A hundred microgram protein were loaded on 12% sodium dodecyl sulfate-polyacrylamide gel electrophoresis (SDS-PAGE) and transferred to polyvinylidene difluoride (PVDF) membranes (Sigma-Aldrich, USA). Membranes were blocked in 5% w/v bovine serum albumin (BSA) for 1 h at room temperature. Then membranes were incubated at 4°C overnight with the following primary antibodies: anti-TRAIL (Cell Signaling Technology, USA, 1:1,000), anti-FasL (Absin, China, 1:500), and anti-β-actin (Santa Cruz Biotechnology, USA, 1:1,000). β-actin was used as the internal control. Then the blot was incubated with the corresponding IRDye 800CW-conjugated secondary antibody (LI-COR Biosciences, USA, 1:10,000) for 1 h at room temperature. Signals were detected using Odyssey Infrared Imaging System (LI-COR Biosciences, USA) and the blots were quantified using ImageJ (McMaster Biophotonics Facility, Canada).

### Immunohistochemistry

Immunohistochemistry was performed as previously described (24). Four micrometer sections of formalin-fixed paraffin-embedded decidual samples were incubated overnight with anti-FasL antibody (1:200; Abcam, USA) and anti-TRAIL antibody (1:200; Cell Signaling Technology, USA, 1:500) at 4°C. Monoclonal or polyclonal rabbit IgG served as the negative control. Bright-field images were taken using Leica DM2500 (Leica, Germany). Images were randomly taken from each section, and the average optic density was identified with ImageJ (McMaster Biophotonics Facility, Canada).

### Statistical Analysis

Results are presented as mean ± standard deviation (SD). Unpaired Student's *t*-test was used to analyze the differences between the two groups. When the variances of the two groups differed in F test, the Mann-Whitney *U*-test was used to compare the two groups. Comparison among multiple groups was carried out by one-way ANOVA followed by Tukey's *post-hoc* test. Correlations between parameters were evaluated using Pearson correlation analysis. *P*-value < 0.05 was considered to be statistically significant. All statistical analyses were performed using GraphPad Prism 7 Software (GraphPad Software, USA).

## Results

### PMN-MDSC Was the Major Subset of Decidual MDSC and Decreased in the URPL Group

**Figure 1:**
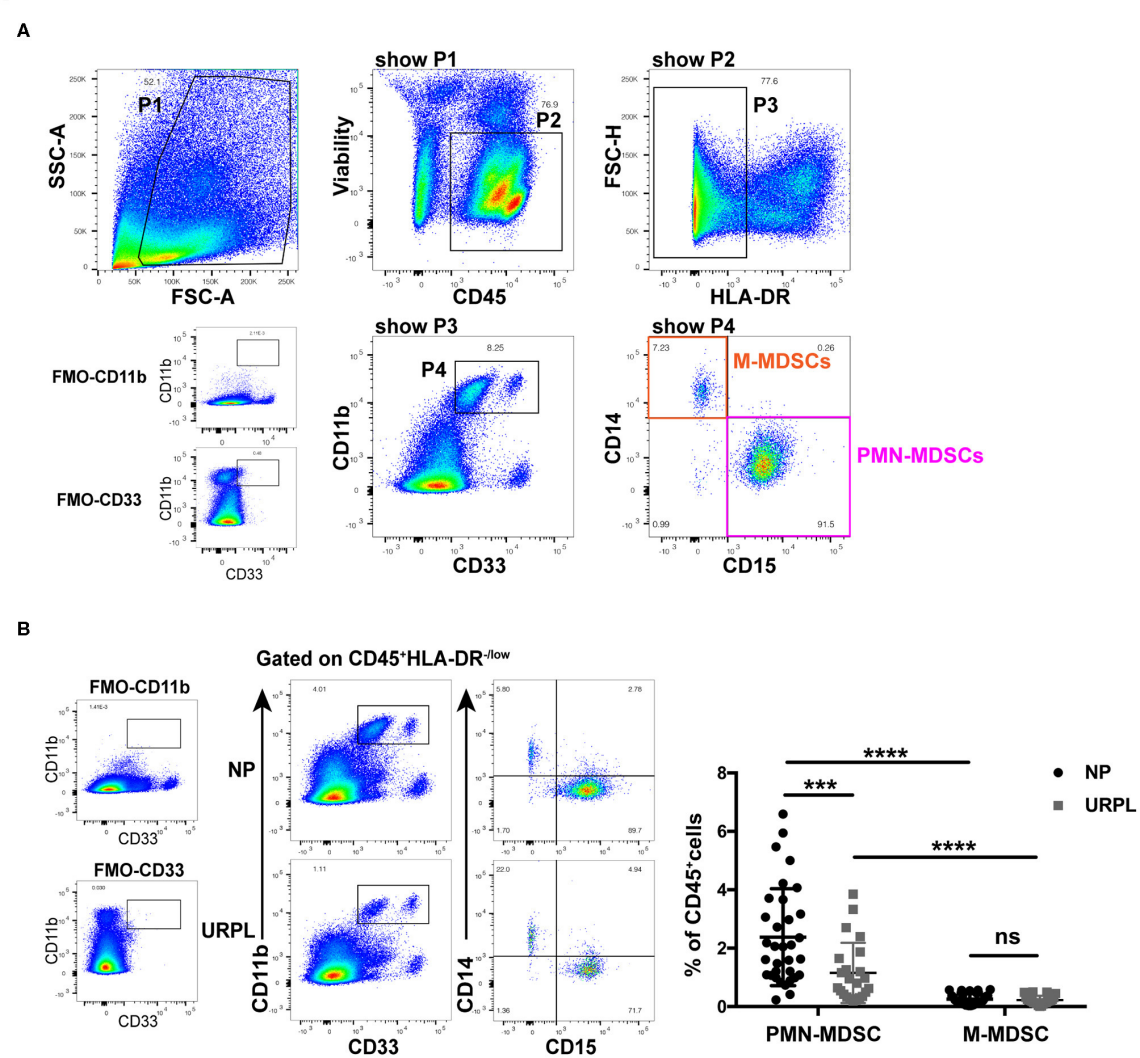
Phenotypic characteristics and frequency of MDSC subsets of human decidual tissue in the normal pregnancy (NP) group and the unexplained recurrent spontaneous abortion (URPL) group. **(A)** Representative flow cytometry showing gating strategy of PMN-MDSC (gated on HLA-DR^−/low^CD11b^+^CD33^+^CD15^+^CD14^−^) and M-MDSC (gated on HLA-DR^−/low^CD11b^+^CD33^+^CD15^−^CD14^+^) in decidua. **(B)** Representative flow cytometry of decidual PMN-MDSC and M-MDSC of the NP and the URPL group (left). Percentage of decidual PMN-MDSC and M-MDSC of NP (*n* = 33) and URPL (*n* = 23) (right) were analyzed (mean ± SD, Mann-Whitney *U*-test). ***P < 0.001; ****P < 0.0001; ns, not significant. FMO, Fluorescent minus one.

We used current consensual marker combinations for characterization of MDSC subsets. PMN-MDSC were defined as HLA-DR^−/low^CD11b^+^CD33^+^CD15^+^CD14^−^ and M-MDSC were defined as HLA-DR^−/low^CD11b^+^CD33^+^CD15^−^CD14^+^ (Figure 1A). We found that both CD33^dim^CD15^+^ PMN-MDSC and CD33^bright^CD14^+^ M-MDSC existed in decidua of early pregnancy. To determine the potential role of MDSC in pathogenesis of URPL, we analyzed percentage of MDSC subsets within the total CD45^+^ leukocytes of 23 patients with URPL and 33 women with normal pregnancy using flow cytometry. Compared with M-MDSC, more PMN-MDSC accumulated in decidua in both the NP group and the URPL group (Figure 1B; *P* < 0.0001). Notably, only decidual PMN-MDSC significantly decreased in the URPL group compared with normal pregnancy (Figure 1B; *P* = 0.001).

### Decidual PMN-MDSC in Both the NP Group and the URPL Group Had Suppressive Activity

**Figure 2:**
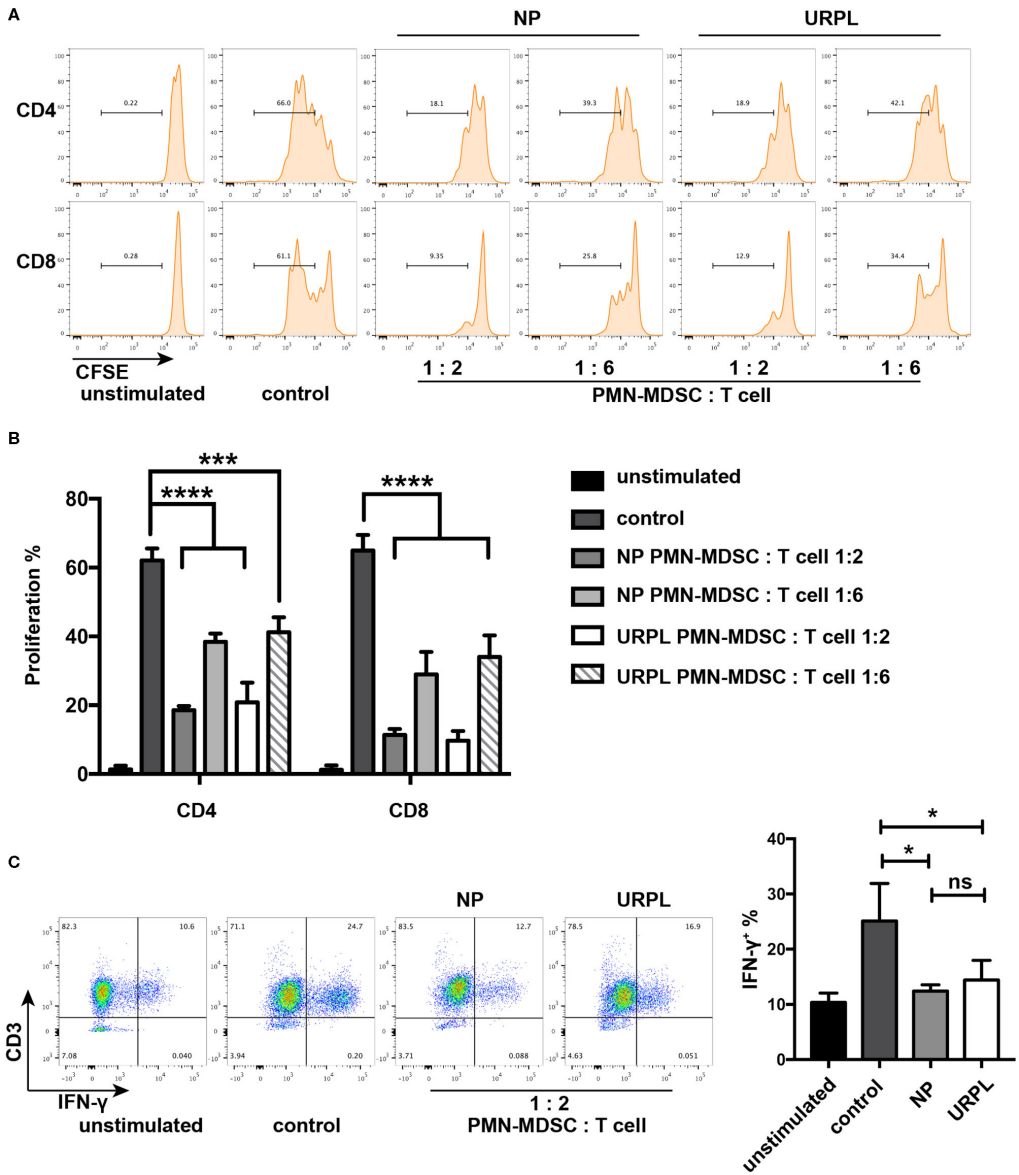
Functional characteristics of decidual PMN-MDSC in the NP group and URPL group. **(A)** and **(B)** CD3/CD28-stimulated T cells were cocultured with purified PMN-MDSC of the NP group and the URPL group from decidua of pregnancy between 6 and 9 weeks at a ratio of 2:1 or 6:1 for 3.5 days. The percentage of proliferative CD4^+^ T cells or CD8^+^ T cells were analyzed (mean ± SD, one-way ANOVA, Tukey's *post-hoc* test). **(C)** CD3/CD28-stimulated T cells were cocultured with purified PMN-MDSC of the NP group and the URPL group from decidua of pregnancy between 6 and 9 weeks at a ratio of 2:1 for 3.5 days. Percentage of IFN-γ-expressing T cells were analyzed (mean ± SD, one-way ANOVA, Tukey's *post-hoc* test). *n* = 3; *P < 0.05; ***P < 0.001; ****P < 0.0001; ns, not significant.

T cell suppression ability is a hallmark of MDSC and is indispensable when defining MDSC. PMN-MDSC were isolated from decidual tissues of the NP group and the URPL group and then cocultured with purified CD3^+^ T cells at ratio of 1:2 or 1:6 in the presentence of anti-CD3/CD28 stimulation for 3.5 days. PMN-MDSC in both groups remarkably suppressed proliferation of CD4^+^ T cells or CD8^+^ T cells (Figures 2A,B). IFN-γ production was also suppressed by PMN-MDSC in both the NP group (Figure 2C; *P* = 0.02) and the URPL group (Figure 2C; *P* = 0.04). Altogether, these data indicated that PMN-MDSC in both the NP group and URPL group exerted potent suppression ability.

### Decidual PMN-MDSC in the URPL vs. the NP Groups Showed Different Gene and Pathway Signature

**Figure 3:**
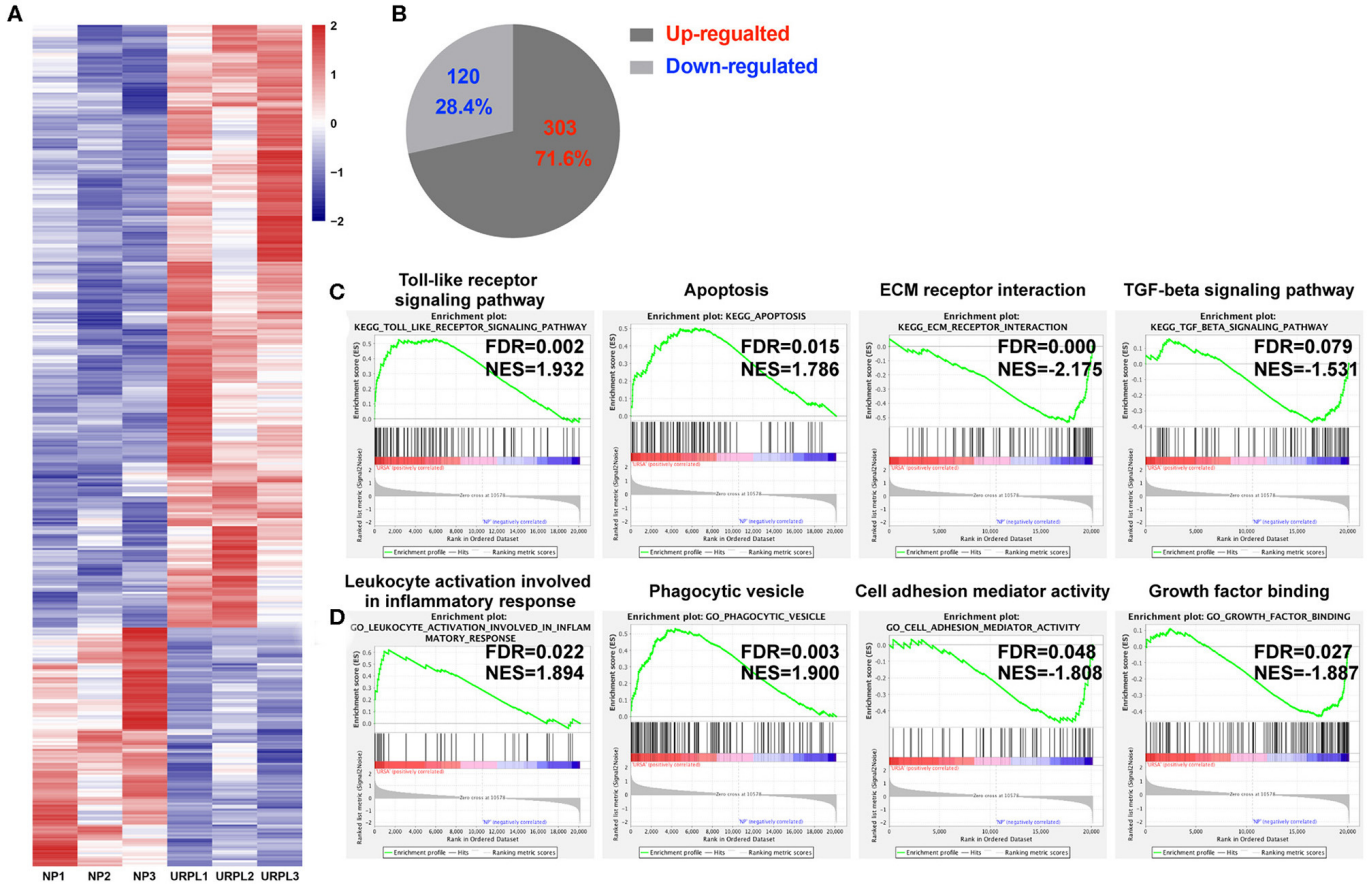
Differential expression genes and pathways in human decidual PMN-MDSC of the NP and the URPL group. **(A)** Heatmap showing differentially expressed genes (DEGs). Rows in the heatmap represent DEGs. **(B)** The number and proportion of DEGs in PMN-MDSC that were upregulated or downregulated in the URPL group (*n* = 3) vs. the NP group (*n* = 3). **(C)** GSEA analysis of KEGG gene sets including toll-like receptor signal pathway, apoptosis, ECM receptor interaction and TGF-beta signaling pathway. **(D)** GSEA analysis of GO gene sets including leukocyte activation involved in leukocyte activation involved in inflammatory response, phagocytic vesicle, cell adhesion mediator activity, and growth factor binding. Normalized enrichment score (NES) reflects the degree to which a gene set was upregulated (positive NES) or downregulated (negative NES) in PMN-MDSC of URPL and false discovery rate (FDR) represents statistical significance of difference.

To evaluate the potential role of PMN-MDSC in maintaining the normal pregnancy, we performed whole-genome expression profile analysis of decidual PMN-MDSC in the NP group and the URPL group. The gene expression pattern of the decidual PMN-MDSC was significantly different between the NP group and the URPL group. Altogether, 423 differentially expressed genes (DEG) exhibited a fold change of >1.5 with an adjusted *P*-value of < 0.05; 303 genes were upregulated and 120 genes were down-regulated in the URPL group compared with the NP group (Figures 3A,B). To elucidate functional features of PMN-MDSC in NP and URPL, GSEA were performed. KEGG gene sets and GO gene sets were used in the analysis. Of note, toll-like receptor signal pathway, apoptosis, leukocyte activation involved in inflammatory response and phagocytic vesicle were significantly enriched in the URPL group while extracellular matrix (ECM) receptor interaction, TGF-beta signaling pathway, cell adhesion mediator activity and growth factor binding were remarkably enriched in the NP group (Figures 3C,D).

### Decidual PMN-MDSC in URPL Underwent More Apoptosis

**Figure 4:**
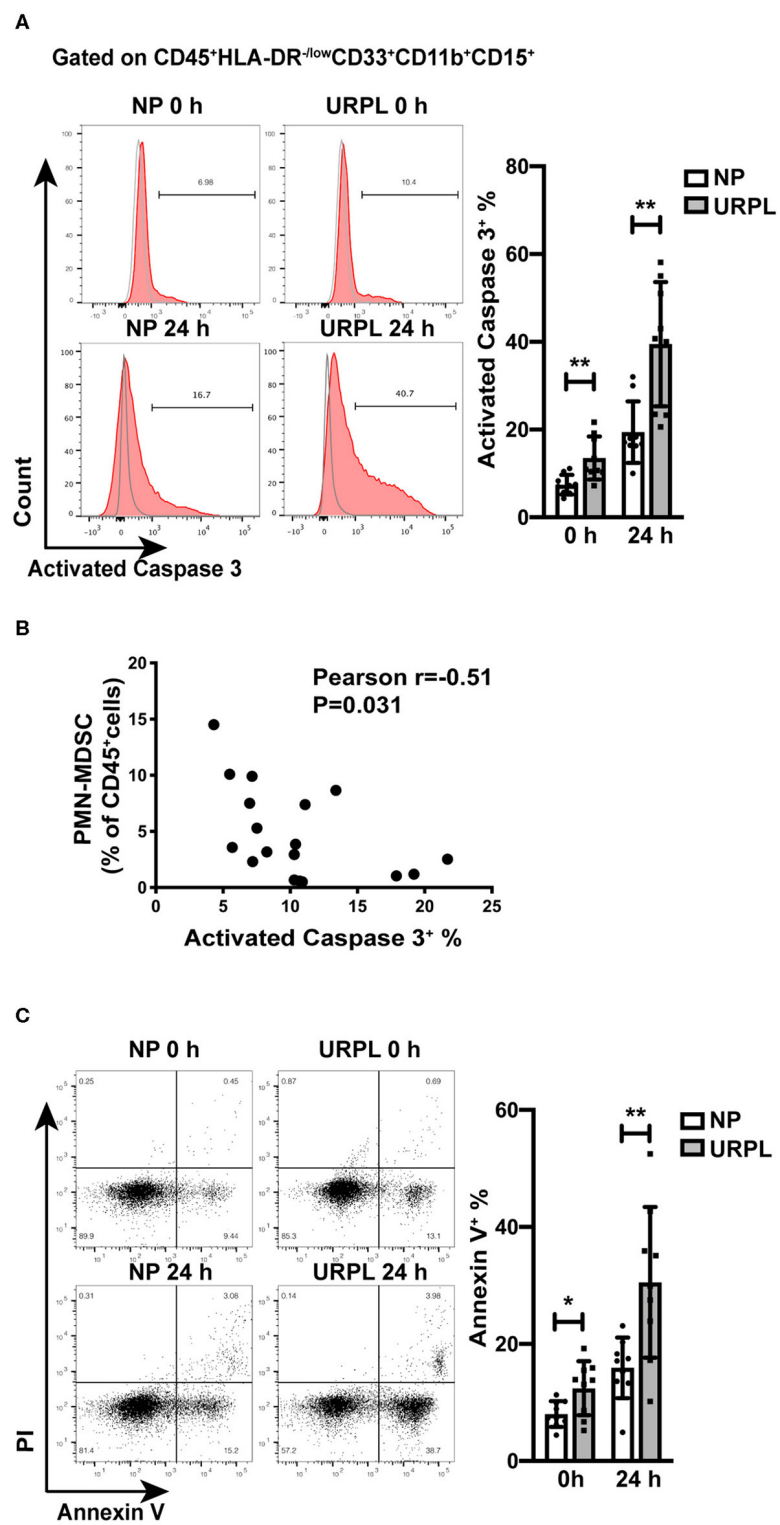
Apoptosis of decidual PMN-MDSC of NP and URPL. **(A)** After isolated from decidual tissues or cultured *in vitro* for 24 h, expression of activated Caspase 3 of decidual PMN-MDSC between the NP group (*n* = 9) and the URPL group (*n* = 9) were determined. **(B)** The correlation of activated Caspase 3 expression in decidual PMN-MDSC with proportion of PMN-MDSC within the total decidual CD45^+^ leukocytes was analyzed (*n* = 18). **(C)** After isolated from decidual tissues or cultured *in vitro* for 24 h, expression of Annexin V in PMN-MDSC between the NP group (*n* = 9) and the URPL group (*n* = 9) were analyzed. Mean ± SD, unpaired Student's *t*-test; *P < 0.05; **P < 0.01.

To further examine whether decidual PMN-MDSC in the URPL group experienced more apoptosis than that in the NP group, we analyzed activated Caspase 3 expression in PMN-MDSC of the two groups. There was significant difference in the proportion of apoptotic cells in freshly isolated decidual PMN-MDSC of the NP group and the URPL group (Figure 4A; *P* = 0.004). After cultured *in vitro* for 24 h, activated Caspase3 expression was also higher in PMN-MDSC of the URPL group (Figure 4A; *P* = 0.002). Furthermore, the proportion of PMN-MDSC within the total decidual CD45^+^ leukocytes was negatively correlated with activated Caspase 3 expression in PMN-MDSC (Figure 4B; Pearson *r* = −0.51, *P* = 0.031). The apoptosis of decidual PMN-MDSC was also examined by Annexin V staining. For PMN-MDSC which were freshly isolated or cultured for 24 h, the percentage of Annexin V^+^ PMN-MDSC was higher in the URPL group compared with the NP group (Figure 4C; *P* = 0.02, *P* = 0.006). Activated Caspase 3 expression was of no difference between the NP group and the URPL group for freshly isolated decidual NK cells (Supplemental Figure 1) and decidual T cells (Supplemental Figure 1). These data indicated that decidual PMN-MDSC in the URPL group underwent more apoptosis than that in the NP group.

### Decidual FasL and TRAIL Expression Were Increased in URPL

**Figure 5:**
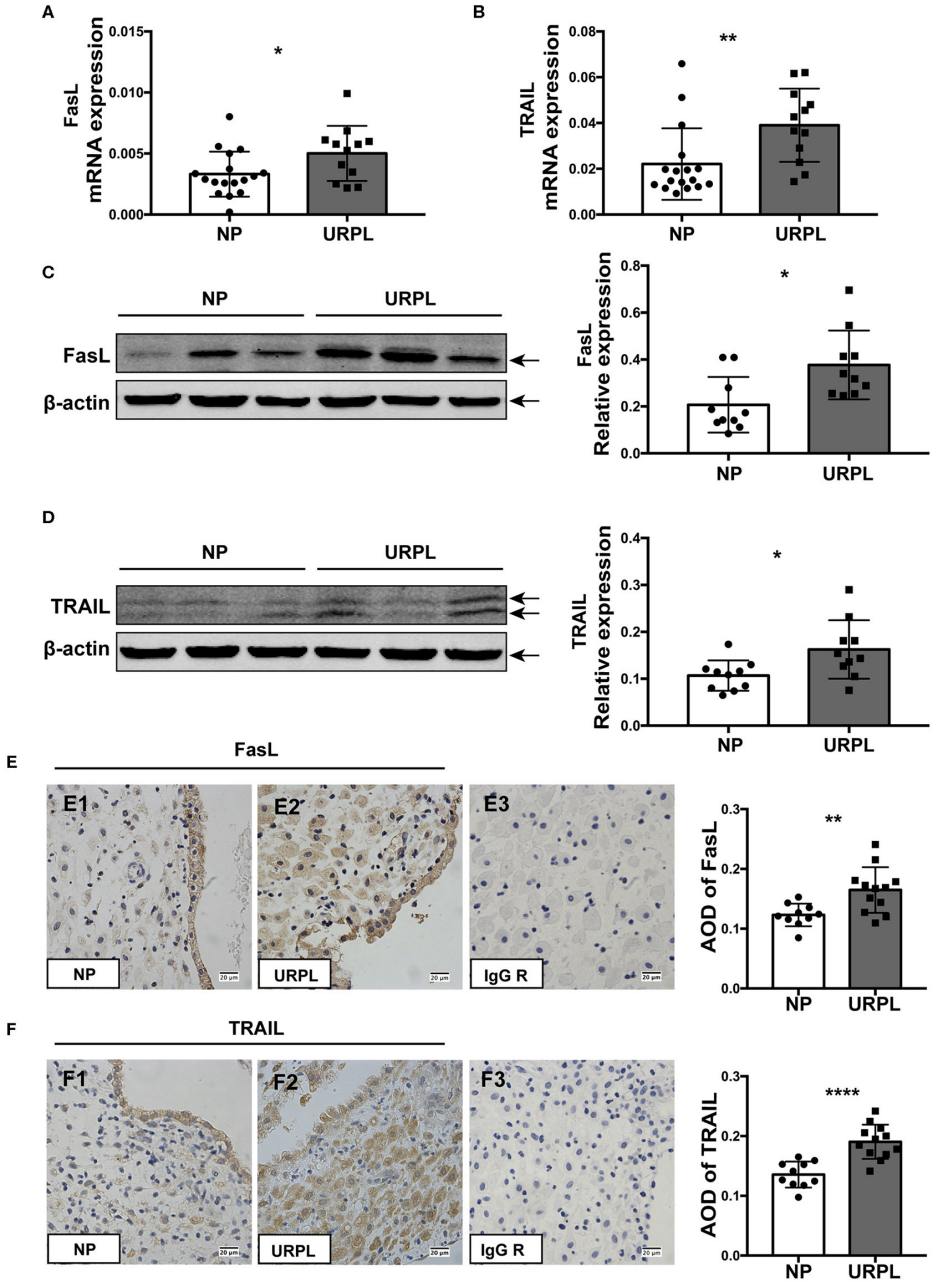
FasL and TRAIL expression in human decidual tissue of the NP and the URPL group. **(A)** and **(B)** Decidual FasL and TRAIL mRNA expression in the NP group (*n* = 17) and the URPL group (*n* = 12) was quantified using 2^−Δ*CT*^ method normalized to GAPDH. **(C,D)** Representative western blot results and statistical analysis of FasL and TRAIL in the NP (*n* = 10) group and URPL (*n* = 10) group were shown. Relative protein amount was normalized to β-actin. Arrows indicate the specific bands for each antibody. **(E,F)** Representative immunohistochemical staining image and quantification of the average optical density (AOD) of FasL and TRAIL in the NP group (*n* = 10) (E1, F1) and the URPL group (*n* = 12) (E2, F2). Polyclonal or monoclonal rabbit IgG substituted primary antibodies in the negative control (E3, F3) Original magnification, ×400; mean ± SD, unpaired Student's *t*-test; *P < 0.05; **P < 0.01; ****P < 0.0001.

We determined the expression levels of FasL and TRAIL in decidual tissues of the URPL and the NP group. qRT-PCR results showed that mRNA level of FasL (Figure 5A; *P* = 0.034) and TRAIL (Figure 5B; *P* = 0.008) significantly increased in the URPL group. Western blot showed protein levels of FasL (Figure 5C; *P* = 0.011) and TRAIL (Figure 5D; *P* = 0.022) were remarkably upregulated in the URPL group. Immunohistochemistry staining showed that in the NP group, FasL expression was stronger in the glandular epithelial cells than that in the decidual stromal cells (Figure 5E1); however, in the URPL group, both glandular epithelial cells and decidual stromal cells showed moderate FasL expression (Figure 5E2). TRAIL was localized in both glandular epithelial cells and decidual stromal cells (Figure 5F1,F2), and epithelial cells showed stronger expression than stromal cells in the NP group (Figure 5F1). FasL and TRAIL staining were stronger in the URPL group (Figures 5E,F; *P* = 0.005, *P* < 0.0001). Localization of glandular epithelial cells and decidual stromal cells were determined by staining of cytokeratin 7 and vimentin (Supplemental Figure 2), respectively. The negative control stained with polyclonal or monoclonal rabbit IgG showed no positive staining (Figure 5E3,F3).

### Apoptosis of Decidual PMN-MDSC Was Regulated by TRAIL and TRAIL-Rs

**Figure 6:**
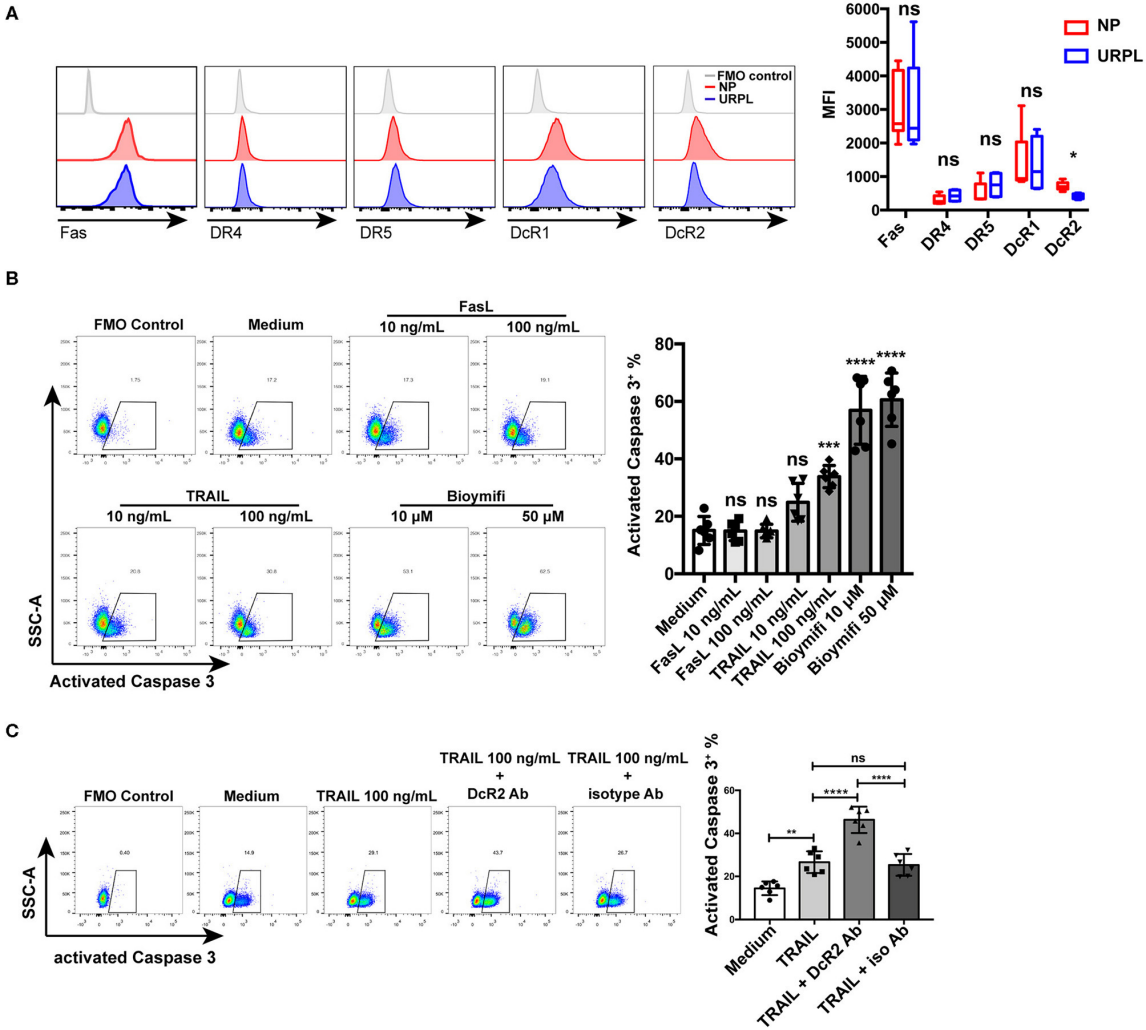
TRAIL and TRAIL-Rs mediated decidual PMN-MDSC apoptosis. **(A)** Representative flow cytometry of Fas, DR4, DR5, DcR1, and DcR2 expression of decidual PMN-MDSC (left) and statistical analysis (right, *n* = 4–9, mean ± SD, Mann-Whitney *U*-test). **(B)** Decidual PMN-MDSC from the NP group were treated with FasL (10, 100 ng/mL), TRAIL (10, 100 ng/mL), or DR5 agonist Bioymifi (10, 50 μM) for 24 h (*n* = 6) and activated Caspase 3 expression was analyzed (mean ± SD, one-way ANOVA, Tukey's *post-hoc* test). **(C)** Decidual PMN-MDSC from the NP group were stimulated with TRAIL (100 ng/mL) in the presentence of 10 μg/mL anti-DcR2 Ab or isotype control for 24 h. Activated Caspase 3 was measured. Representative flow cytometry (left) and quantification (right) were shown (*n* = 6) (mean ± SD, one-way ANOVA, Tukey's *post-hoc* test). *P < 0.05; **P < 0.01; ***P < 0.001; ****P < 0.0001; ns, not significant. FMO, Fluorescent minus one.

We then analyzed the expression of membrane receptors of FasL and TRAIL on decidual PMN-MDSC. Decidual PMN-MDSC in both the NP group and the URPL group showed expression of Fas, DR4, DR5, DcR1, and DcR2 (Figure 6A). The expression level of DR4 was rather low since the fluorescence intensity was very close to the FMO control. PMN-MDSC of the two groups did not differ in the expression of Fas, DR4, DR5, or DcR1; however, DcR2 expression on PMN-MDSC was significantly down-regulated in the URPL group (*P* = 0.016). We next investigated whether decidual PMN-MDSC was sensitive to FasL or TRAIL induced apoptosis. PMN-MDSC from the NP group were treated with recombinant human FasL and TRAIL. FasL could not induce apoptosis of decidual PMN-MDSC at the concentration of both 10 and 100 ng/mL (Figure 6B). Notably, decidual PMN-MDSC showed significantly higher apoptosis (Figure 6B, *P* = 0.0006) after exposure to 100 ng/mL TRAIL. Moreover, exposure to 10 or 50 μM DR5 agonist Bioymifi induced a significantly high level of PMN-MDSC apoptosis than TRAIL (*P* < 0.0001, *P* < 0.0001). To examine whether the response to TRAIL-induced apoptosis was mediated by DcR2 expression, we detected the expression of activated Caspase 3 in decidual PMN-MDSC after exposure of anti-DcR2 Ab and TRAIL for 24 h (Figure 6C). Notably, compared with isotype control, DcR2 blockade significantly increased TRAIL-induced apoptosis (*P* < 0.0001).

## Discussion

As an important regulator of the immune system, the role of MDSC in pregnancy has been established in several studies (5, 6, 13, 14). A certain number of MDSC take part in maintaining immune tolerance during normal pregnancy and a lack of MDSC can lead to pregnancy failure (14, 15). Consistent with previous studies of our group and others (5, 13), we found that PMN-MDSC was the major subset of MDSC in human decidua since the percentage of PMN-MDSC remarkably exceeded that of paired M-MDSC in both the NP group and the URPL group. Also, only PMN-MDSC, but not M-MDSC, decreased significantly in decidua of patients with URPL compared with normal pregnancy. During pregnancy, PMN-MDSC can facilitate maternal-fetal immune tolerance via crosstalk with various immune cells. PMN-MDSC suppress T cell proliferation via ROS or Arginase I and polarize CD4^+^ T cells toward a Th2 cytokine response (4, 14, 15). PMN-MDSC can also induce regulatory T cells in a TGF-beta dependent manner (5). Moreover, PMN-MDSC can inhibit NK cytotoxicity by inhibiting expression of perforin, granzyme B, and NKG2D (6). We found that PMN-MDSC of both the NP group and the URPL group potently suppressed T cell proliferation as well as cytokine production, validating the immune regulatory ability of decidual PMN-MDSC.

According to our whole-genome expression profile analysis, genes upregulated in PMN-MDSC of the URPL group significantly enriched in apoptosis compared with PMN-MDSC of the NP group. Flow cytometry analysis further validated that compared with PMN-MDSC in the NP group, PMN-MDSC in the URPL group underwent more apoptosis. Mechanisms on MDSC apoptosis and survival have been investigated in other microenvironments, however, the results remain controversial. Cytotoxic T cells can induce MDSC apoptosis via Fas/Fas ligand (FasL) signaling pathway while resistance to Fas-mediated apoptosis contributes to the presence of MDSC in tumor, and this effect is exclusive in MDSC since other myeloid cells also express a similar level of Fas but do not respond to FasL (25, 26). Interestingly, in another mouse model, FasL deficiency leads to reduced MDSC and skews MDSC toward M-MDSC, indicating that PMN-MDSC decreases more after FasL knockdown (27). Moreover, inflammation conditions can protect MDSC from extrinsic-induced apoptosis (28). Tumor necrosis factor (TNF)-related apoptosis induced ligand (TRAIL) is another regulator of MDSC apoptosis via interacting with membrane bound TRAIL receptors (TRAIL-Rs). TRAIL-R1 (DR4 or CD261) and TRAIL-R2 (DR5 or CD262) are two death receptors, and ligation of TRAIL with either of them can activate the apoptotic pathway. TRAIL-R3 (DcR1 or CD263) and TRAIL-R4 (DcR2 or CD264) are two decoy receptors which bind to TRAIL without further inducing apoptosis (29). In a murine model, high expression of DR5 mediated TRAIL-induced effects, while in human, differently expressed DcR1 and DcR2 could regulate cell fate of PMN-MDSC, indicating different apoptosis mechanisms between species (19, 20). In primary HIV-infected individuals, high TRAIL level is also associated with decreased PMN-MDSC (30).

FasL and TRAIL are located in placenta as well as decidua, and are important for immune privilege and successful pregnancy since they can mediate apoptosis of cytotoxic T cells or other immune cells with cell toxicity (31, 32). We found that in human decidua both glandular epithelial cells and decidual stromal cells expressed FasL and TRAIL. FasL and TRAIL levels were elevated in the URPL group, which is in accordance with that excessive FasL as well as TRAIL could also be involved in URPL (33–36). Then we detected Fas and TRAIL-Rs levels of decidual PMN-MDSC between the URPL and the NP group and only DcR2 expression was differentially expressed. Decidual PMN-MDSC did not respond to FasL-mediated apoptosis. However, they were sensitive to TRAIL-mediated apoptosis via Caspase-3 dependent pathway. Interestingly, 100 ng/mL TRAIL only increased two times apoptosis while DR5 agonist Bioymifi increased up to four times apoptosis. Further *in vitro* blocking of DcR2 can facilitate TRAIL-induced apoptosis in human decidual PMN-MDSC, which is in line with a clinical trial showing that DR5 agonist selectively eliminated PMN-MDSC in cancer patients and the effect is reversely correlated with DcR2 expression level (20).

This is the first study focusing on decidual PMN-MDSC survival during early human pregnancy. We found that the apoptosis levels of two major decidual leukocytes, NK and T cells, are similar in the NP and the URPL groups, implying that PMN-MDSC apoptosis might not be the result of enhanced apoptosis state in the URPL group. In addition, previous studies mainly focused on disparity in MDSC number between the NP and URPL groups. In the present study, although we showed that PMN-MDSC in both NP and URPL groups exerted potent immune suppressive function, the gene expression profile indicated significantly different enriched biology pathways between PMN-MDSC of the two groups. Genes upregulated in the PMN-MDSC of the URPL group were enriched in toll-like receptor signal pathway, leukocyte activation involved in inflammatory response and phagocytic vesicle, indicating that these cells experienced more inflammation than PMN-MDSC in the NP group. The ECM receptor interaction, TGF-beta signaling pathway, cell adhesion mediator activity and growth factor binding were negatively enriched in PMN-MDSC of the URPL group, indicating impaired interaction with extracellular matrix and other cells, which is important for cell recruitment and immune crosstalk. These issues remain be further investigation.

In conclusion, we demonstrate that human decidual PMN-MDSC in URPL are more sensitive to TRAIL-mediated apoptosis signal pathway owing to elevated TRAIL and decreased DcR2 expression. This could be a mechanism of impaired viability of decidual PMN-MDSC in URPL, however, the underlying molecular pathways of decidual PMN-MDSC apoptosis needs to be further elucidated. The observations presented in this study provide a new insight into mechanisms of dysregulation of PMN-MDSC in URPL, and therapeutic targeting on TRAIL-induced apoptosis signaling may provide novel strategies for URPL treatment.

## Data Availability Statement

The datasets generated for this study can be found in the Gene Expression Omnibus - GSE139180.

## Ethics Statement

The studies involving human participants were reviewed and approved by Human Research Ethics Committee of Renji Hospital. The patients/participants provided their written informed consent to participate in this study.

## Author Contributions

CL, XZ, and AZ conceived and designed this study. CL and XZ performed the experiments. CL, XZ, CC, FG, and QW collected the samples. CL, XZ, and XK analyzed the data. CL drafted the manuscript. AZ revised the manuscript. All authors contributed to the article and approved the submitted version.

## Conflict of Interest

The authors declare that the research was conducted in the absence of any commercial or financial relationships that could be construed as a potential conflict of interest.
