# Supplementary material for: Upregulated TRAIL and Reduced DcR2 Mediate Apoptosis of Decidual PMN-MDSC in Unexplained Recurrent Pregnancy Loss
Source: Front Immunol. 2020 Jun 30;11:1345. doi: 10.3389/fimmu.2020.01345 (PMC7338483; doi:10.3389/fimmu.2020.01345)
Supplement: Supplementary file 1 [file Data_Sheet_1.docx]

**Supplemental Table S1. Clinical characteristics of women included in MDSCs subsets analysis**

|  | **NP (n=33)** | **URPL (n=23)** | ***P*** |
| --- | --- | --- | --- |
| Age (years) | 29.88±6.01 | 32.13±5.89 | 0.1702^a^ |
| Gestation age (weeks) | 7.55±0.94 | 8.04±0.93 | 0.0549^a^ |
| Previous pregnancy loss | 0 | 2-5 | - |
| Parity | 1-3 | 0 | - |
| Proportion of IVF | 0 | 43.48% (10/23) | - |

NP: normal pregnancy

URPL: unexplained recurrent pregnancy loss

^a^ Unpaired Student’s t test.

**Supplemental Table S2. Primers used for real-time quantitative RT-PCR**

| **Genes** | **Primers** |
| --- | --- |
| **FasL** | Forward: 5’TGCCTTGGTAGGATTGGGC 3’ |
|  | Reverse: 5’GCTGGTAGACTCTCGGAGTTC 3’ |
| **TRAIL** | Forward: 5’TGCGTGCTGATCGTGATCTTC 3’ |
|  | Reverse: 5’GCTCGTTGGTAAAGTACACGTA 3’ |
| **GAPDH** | Forward: 5’CAGGAGGCATTGCTGATGAT 3’ |
|  | Reverse: 5’GAAGGCTGGGGCTCATTT 3’ |

**Supplemental Figure S1.**

Cytokeratin 7 (CK7) stained for epithelial cells and Vimentin stained for stromal cells.

**Supplemental Figure S2.**

(A) and (B) Activated caspase 3 expression in T cells (CD45^+^CD3^+^) and NK cells (CD45^+^CD3^-^CD16^-^CD56^bright^) after isolated from decidual tissue were determined by flow cytometry between the NP group (n=5) and the URPL group (n=5).
