# Supplementary figures and images for: Upregulated TRAIL and Reduced DcR2 Mediate Apoptosis of Decidual PMN-MDSC in Unexplained Recurrent Pregnancy Loss
Source: Front Immunol. 2020 Jun 30;11:1345. doi: 10.3389/fimmu.2020.01345 (PMC7338483; doi:10.3389/fimmu.2020.01345)

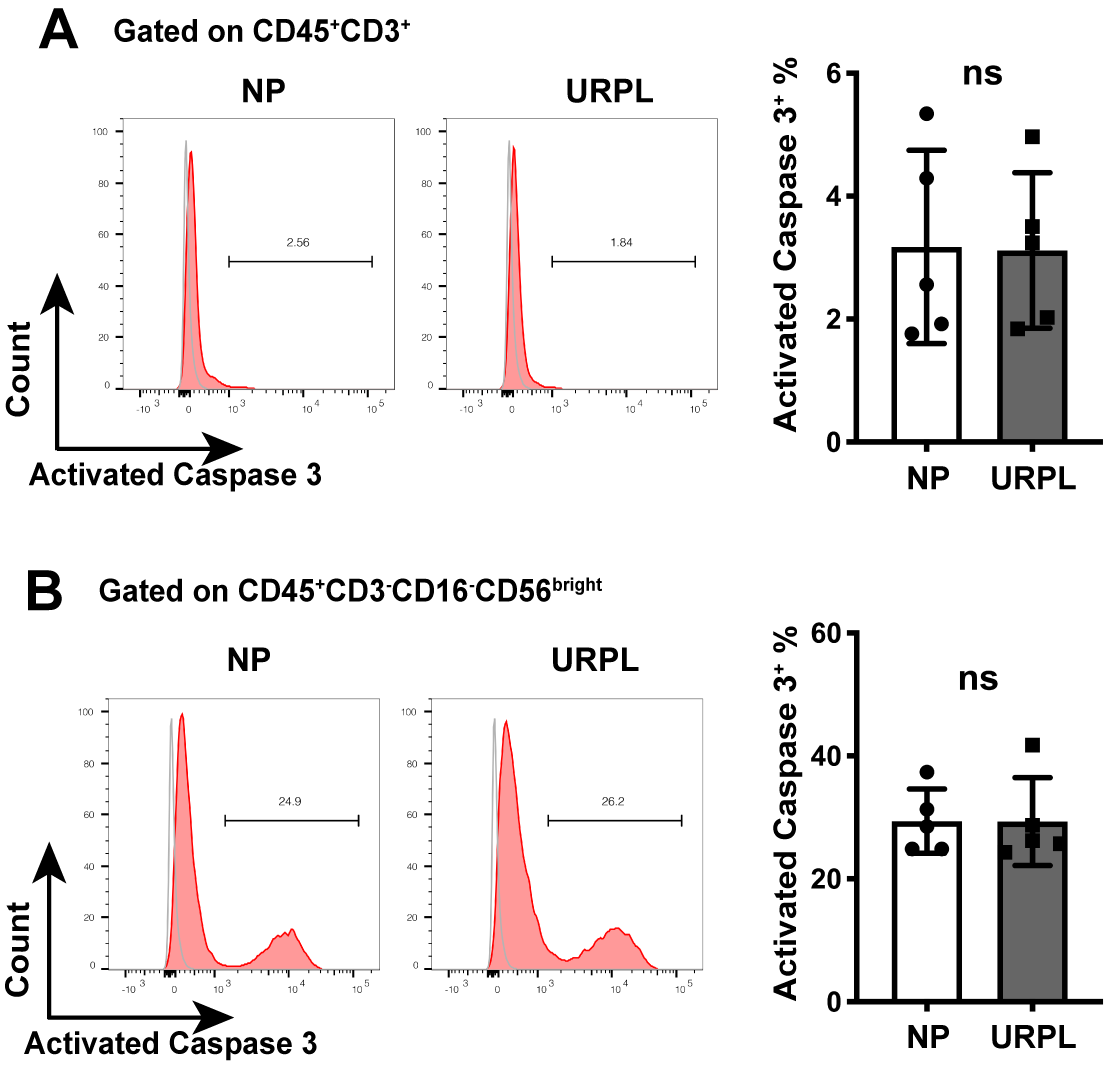

Supplement: Supplementary file 2 [file Image_1.TIF]

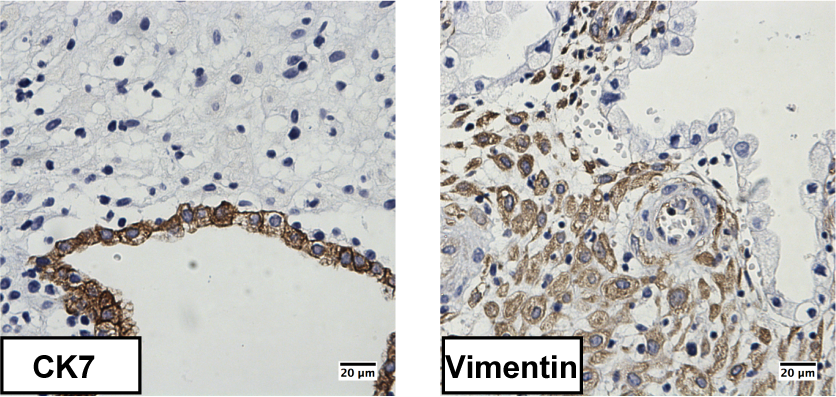

Supplement: Supplementary file 3 [file Image_2.TIF]
